# Evaluation of Bacteriophage-Antibiotic Combination Therapy for Biofilm-Embedded MDR *Enterococcus faecium*

**DOI:** 10.3390/antibiotics11030392

**Published:** 2022-03-15

**Authors:** Katherine Lev, Ashlan J. Kunz Coyne, Razieh Kebriaei, Taylor Morrisette, Kyle Stamper, Dana J. Holger, Gregory S. Canfield, Breck A. Duerkop, Cesar A. Arias, Michael J. Rybak

**Affiliations:** 1 Anti-Infective Research Laboratory, College of Pharmacy and Health Sciences, Wayne State University, Detroit, MI 48201, USA; katlev@wayne.edu (K.L.); ashlan.kunzcoyne@wayne.edu (A.J.K.C.); r.kebriae@wayne.edu (R.K.); kyle.stamper@wayne.edu (K.S.); dholger@wayne.edu (D.J.H.); 2 Department of Pharmacy and Clinical Services, Medical University of South Carolina College of Pharmacy, Charleston, SC 29208, USA; morritay@musc.edu; 3 Department of Immunology and Microbiology, University of Colorado School of Medicine, Aurora, CO 80045, USA; canfielg@stanford.edu (G.S.C.); breck.duerkop@cuanschutz.edu (B.A.D.); 4 Department of Infectious Diseases, University of Colorado School of Medicine, Aurora, CO 80045, USA; 5 Division of Infectious Diseases, Houston Methodist Hospital, Houston, TX 77030, USA; caarias@houstonmethodist.org; 6 Center for Infectious Diseases Research, Houston Methodist Research Institute, Houston, TX 77030, USA; 7 School of Medicine, Wayne State University, Detroit, MI 48201, USA

**Keywords:** bacteriophage, phage therapy, *Enterococcus faecium*, biofilm, antimicrobial, frequency of resistance, phage sensitivity, resistance management, nontraditional antibacterial

## Abstract

Multidrug-resistant (MDR) *Enterococcus faecium* is a challenging pathogen known to cause biofilm-mediated infections with limited effective therapeutic options. Lytic bacteriophages target, infect, and lyse specific bacterial cells and have anti-biofilm activity, making them a possible treatment option. Here, we examine two biofilm-producing clinical *E. faecium* strains, daptomycin (DAP)-resistant R497 and DAP-susceptible dose-dependent (SDD) HOU503, with initial susceptibility to *E. faecium* bacteriophage 113 (ATCC 19950-B1). An initial synergy screening was performed with modified checkerboard MIC assays developed by our laboratory to efficiently screen for antibiotic and phage synergy, including at very low phage multiplicity of infection (MOI). The data were compared by one-way ANOVA and Tukey (HSD) tests. In 24 h time kill analyses (TKA), combinations with phage-DAP-ampicillin (AMP), phage-DAP-ceftaroline (CPT), and phage-DAP-ertapenem (ERT) were synergistic and bactericidal compared to any single agent (ANOVA range of mean differences 3.34 to 3.84 log_10_ CFU/mL; *p* < 0.001). Furthermore, phage-DAP-AMP and phage-DAP-CPT prevented the emergence of DAP and phage resistance. With HOU503, the combination of phage-DAP-AMP showed the best killing effect, followed closely by phage-DAP-CPT; both showed bactericidal and synergistic effects compared to any single agent (ANOVA range of mean differences 3.99 to 4.08 log_10_ CFU/mL; *p* < 0.001).

## 1. Introduction

Vancomycin-resistant enterococci (VRE) pose major therapeutic challenges due to their high prevalence in nosocomial infections, coupled with their propensity to develop resistance to standard-of-care (SOC) antibiotics [1]. Infections caused by VRE are endemic to hospitals, carry a high degree of morbidity and mortality, and prolong hospital length of stay, making them a rising public health threat [2,3,4,5]. Of note, *E. faecium* infections exhibit higher rates of antibiotic resistance, have increased mortality rates, and result in significantly higher hospital costs compared to *E. faecalis* [3,6]. Clearly, there is an urgent need for innovative treatment options for serious VRE infections [3,6,7].

A biofilm is a multilayer, three-dimensional aggregate of microbial cells, extracellular DNA, and proteins, bound by a polysaccharide matrix, which provides biofilm stability and serves as a bacterial shield against antimicrobials and immune modulators [8,9,10,11,12]. This bacterial structure complicates therapeutic decisions, since the selected treatment should be effective within the bacterial biofilm. *E. faecium* strains have a predisposition to colonize indwelling medical devices and form biofilms, which contribute to their ability to withstand antibiotics and evade the host immune system, allowing their persistence during infection and, worse, treatment failure [12,13,14].

Daptomycin (DAP) is a concentration-dependent cyclic lipopeptide with bactericidal activity against VRE in vitro by disrupting cytoplasmic membranes and peptidoglycan synthesis [15,16,17]. Furthermore, DAP can rapidly penetrate inside biofilms, as previously reported with fluorescent visualization [18]. However, the recent emergence of DAP-resistant VRE isolates due to substitutions in the *liaFSR* regulatory system points to an urgent need for viable options given the limited therapeutic alternatives [19,20,21,22,23,24,25].

Lytic bacteriophages (phages) are viruses that may serve as a novel therapy against biofilm-mediated infections given their ability to target specific host organisms and replicate within bacteria leading to an abundance of phage at the site of infection [26,27,28,29]. Furthermore, some phages have demonstrated success against biofilms formed by other bacterial pathogens implicated in biofilm-mediated infections [9,30,31,32,33]. The adjunctive use of phage and antibiotic therapies may be more effective than either one alone due to enhanced bactericidal activity and the potential to decrease/eliminate/delay the emergence of antibiotic and/or phage resistance or to re-sensitize resistant strains [34,35,36]. The additional benefit of phage-antibiotic combinations compared to the use of either agent alone has been demonstrated previously with promising results, whereas the use of phage monotherapy resulted in the emergence of phage resistance, which required either adjusting the concentration of the administered phage or the use of phage and antibiotic combinations [30,37,38,39,40,41]. *E. faecium*-specific phage 113 has demonstrated activity against multiple antibiotic-resistant isolates in the planktonic state [39]. This observation led us to investigate the hypothesis that this phage, in combination with antibiotics, has promising activity against *E. faecium* biofilms. The work described here determines: (i) the activity of bacteriophage 113 in combination with DAP and various β-lactams (e.g., ampicillin (AMP), ceftaroline (CPT), or ertapenem (ERT)) against biofilm-producing *E. faecium* isolates; and (ii) the impact of phage presence on both antibiotic and phage resistance in the biofilm state.

## 2. Results

### 2.1. Bacterial Isolates

*E. faecium* clinical isolates, R497 (DAP-resistant, MBIC = 16 µg/mL) and HOU503 (vancomycin-resistant; DAP-susceptible dose dependent (SDD), MBIC = 2 µg/mL), were evaluated in this study due to their initial susceptibility to phage 113 and relatively high biofilm production compared to the other isolates determined by the modified small drop agar method and crystal violet microtiter plate biofilm quantification assay, respectively (Table 1) [42,43,44].

### 2.2. Checkerboard Analyses

**Figure 1:**
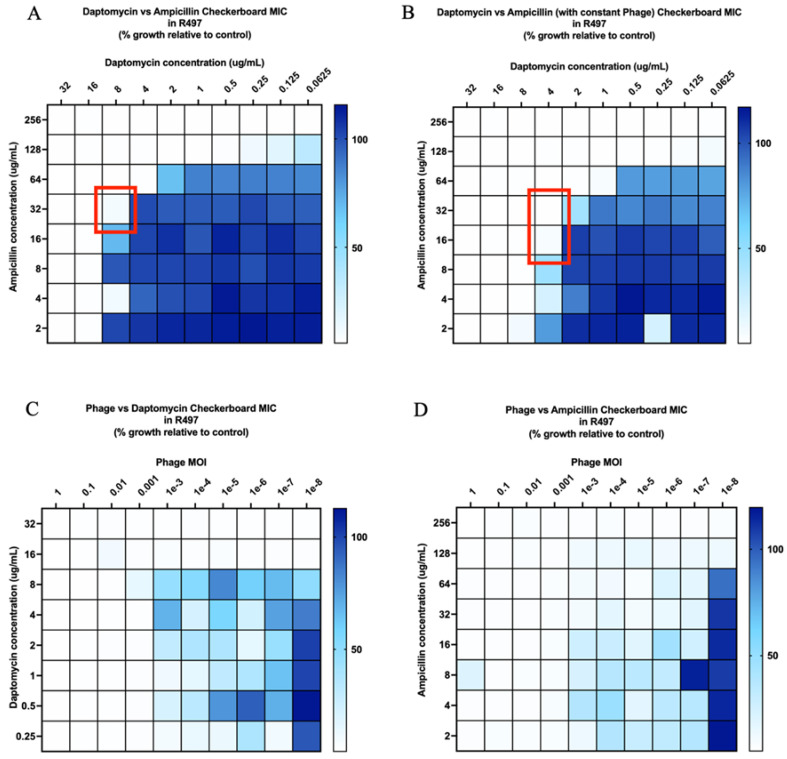
(**A**–**D**) Checkerboard analysis of *E. faecium* strain R497. Additivity, defined as an FIC index >0.5 but <4, is indicated by the red outline in (**A**). Synergy, defined as an FIC index ≤ 0.5, is indicated by the red outline in (**B**). Comparisons are versus growth control and depicted by the blue color gradient as percentage of growth.

**Figure 2:**
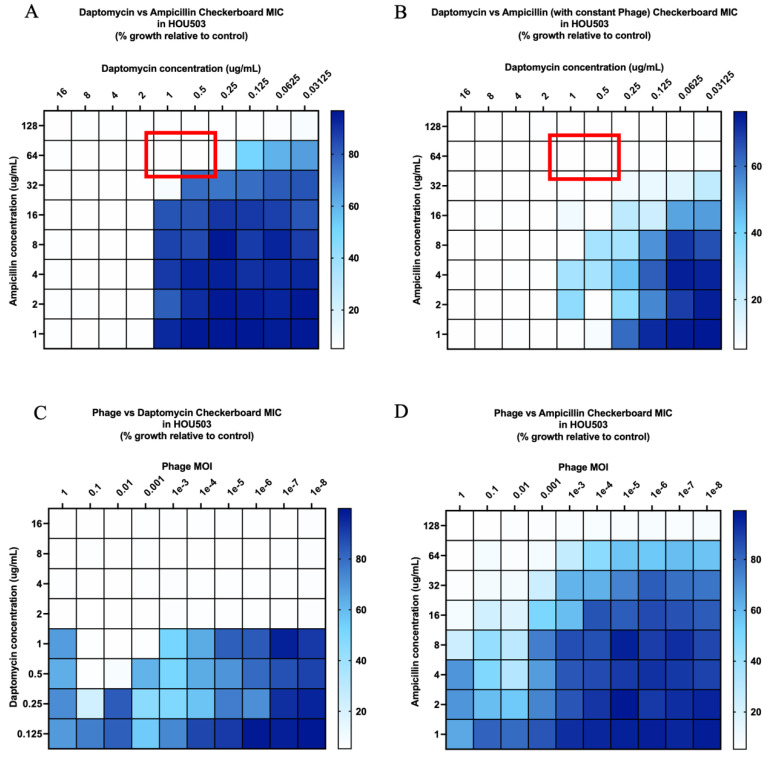
(**A**–**D**) Checkerboard analysis of *E. faecium* strain HOU503. Additivity, defined as an FIC index > 0.5 but <4, is indicated by the red outline in Figure 1A,B. Comparisons are versus growth control and depicted by the blue color gradient as percentage of growth.

DAP in combination with AMP against DAP-resistant R497 in biofilm was additive, with a fractional inhibitory concentration (FIC) index of 1 (Figure 1A), while DAP plus AMP in the presence of subinhibitory phage (MOI of 10^−8^) was synergistic, with an FIC index of 0.5 (Figure 1B). Notably, DAP or AMP in the presence of low phage (MOI of 10^−8)^ showed a 64-fold reduction in comparison to DAP or AMP without phage (Figure 1C,D). Furthermore, the inhibition of R497 growth was greater with the combination of phage plus AMP compared to phage plus DAP. For DAP-SDD HOU503, both phage-DAP-AMP and DAP-AMP combinations were additive, but not synergistic, despite increased killing at lower antibiotic concentrations with the addition of phage (Figure 2A,B; MOI of 10^−8^).

### 2.3. Time-Kill Analyses

**Figure 3:**
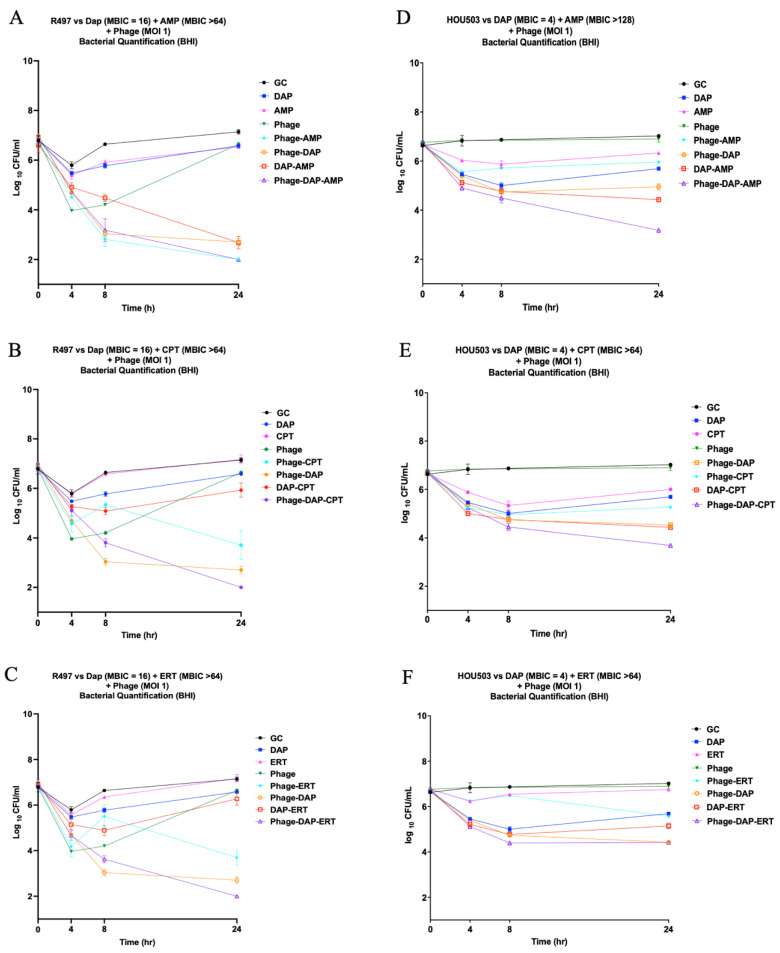
(**A**–**F**) Time-kill analysis of DAP-resistant and DAP-SDD *E. faecium* strains R497 and HOU503, respectively. GC, growth control. (**A**) R497 vs. DAP (0.5xMIC) + AMP (free peak concentration = 72) + phage (MOI = 1). (**B**) R497 vs. DAP (0.5xMIC) + CPT (free peak concentration = 13.2) + phage (MOI = 1) (**C**) R497 vs. DAP (0.5xMIC) + ERT (free peak concentration = 15.5) + phage (MOI = 1) (**D**) HOU503 vs. DAP (0.5xMIC) + AMP (free peak concentration = 72) + phage (MOI = 1) (**E**) HOU503 vs. DAP (0.5xMIC) + CPT (free peak concentration = 13.2) + phage (MOI = 1) (**F**) R497 vs. DAP (0.5xMIC) + ERT (free peak concentration = 15.5) + phage (MOI = 1). DAP, daptomycin; AMP, ampicillin; CPT, ceftaroline; ERT, ertapenem.

In 24 h TKA against DAP-resistant R497, all the combinations of phage-DAP-AMP, phage-DAP-CPT, and phage-DAP-ERT were bactericidal, as compared to any single agent, and exhibited detection-level killing by 24 h (Figure 3A–C). Of note, combination therapy with phage-AMP also showed killing to detection limit (2 log_10_ CFU/mL) at 24 h (Figure 3A). In DAP-SDD HOU503, the combination of phage-DAP-AMP showed the best killing effect with a 3.61 log_10_ CFU/mL reduction from the initial inoculum (Figure 3D), followed closely by phage-DAP-CPT, with a 3.10 log_10_ CFU/mL reduction (Figure 3E), and both were again bactericidal and synergistic compared to the single-agent antibiotic or phage.

### 2.4. Bacteriophage and Antibiotic Resistance Testing

Bacteriophage treatment-emergent resistance was observed for R497 in treatments with phage alone and phage-ERT (Table 2) In HOU503, we observed resistance in all combinations of phage plus single antibiotic, as well as in phage-DAP-ERT, which is consistent with the results of the TKA, which showed minimal killing activity in this combination. However, combinations of phage-DAP-AMP and phage-DAP-CPT prevented the emergence of phage resistance in HOU503. There was no emergence of DAP resistance noted in any of the treatments for R497 or HOU503.

## 3. Discussion

We developed an effective checkerboard MIC method modified from standard MIC testing to screen for antibiotic and phage synergy and identify optimal concentrations of each to best prevent bacterial growth, including at very-low-phage MOI. While the modified checkerboard MIC method provides information pertaining to the inhibition of bacterial growth, it is not meant to measure bacterial killing, and it does provide information on antibiotic effects over multiple time points within a 24-hour period, as seen with TKAs. However, our use of the modified checkerboard MIC method in this study facilitated the accurate and higher throughput screening of effective phage and antibiotic combinations at specific concentrations and phage MOI to then use in TKAs. The data from our modified checkerboard MICs were successful at predicting the TKA results, as demonstrated by multiple phage and antibiotic combinations. For example, in modified checkerboard MIC against R497, DAP-AMP displayed additive activity with an FIC index of 1, and phage-DAP-AMP demonstrated synergy with an FIC index of ≤0.5. In TKA, both DAP-AMP and phage-DAP-AMP combinations demonstrated synergistic activity; however, phage-DAP-AMP achieved killing to the detection limit, while DAP-AMP did not, aligning with the additional killing demonstrated in modified checkerboard MIC by phage-DAP-AMP compared to DAP-AMP. The close alignment between the modified checkerboard MIC data and the TKA results was also demonstrated by other combinations, including phage-AMP and phage-DAP combinations against R497, further validating the use of this method to screen for antibiotic and phage killing effects and possible synergy.

Additionally, we described conserved killing across several different DAP-β-lactam-phage combinations, even when the DAP-β-lactam combinations alone failed against DAP-resistant and DAP-SDD clinical *E. faecium* strains, R497 and HOU503, respectively. As shown previously, DAP plus β-lactam combinations have demonstrated bactericidal activity for some *E. faecium* strains while at the same time demonstrating no activity against others. Thus, our goal was to explore the fundamental questions of whether adjunctive phage administration results in antibiotic enhancement. Here, we demonstrated that in 24-hour biofilm TKA against R497, all the combinations of phage-DAP-AMP, phage-DAP-CPT, and phage-DAP-ERT were synergistic and bactericidal compared to any single agent. We previously demonstrated the significant activity of DAP plus AMP against R497 in the planktonic state, while DAP plus ERT or CPT previously showed no activity [45,46]. Notably, the addition of subinhibitory concentrations of phage to AMP had significant activity against DAP-resistant R497 compared to either phage or AMP alone, thus highlighting this combination for further studies. For HOU503, the combination of phage-DAP-AMP and DAP-AMP was additive, but not synergistic, despite increased killing at lower antibiotic concentrations with the addition of phage at an MOI of 10^−8^. This led to the hypothesis that phage 113 may enhance antibiotic activity in HOU503, but to a lesser extent than in R497.

Furthermore, combinations of phage-DAP-AMP and phage-DAP-CPT prevented treatment-emergent antibiotic and phage resistance in R497, while bacteriophage resistance was observed with R497 in treatments with phage alone and phage-ERT. These data indicate that the addition of DAP, CPT, or AMP to bacteriophage 113 prevents treatment-emergent bacteriophage resistance. As shown previously in the planktonic state [36], the bacterial strain with greater phage susceptibility (R497) showed less emergence of bacteriophage resistance compared to a strain with lower phage susceptibility (HOU503), although an analysis of the emergence of resistance over a longer period may be necessary to confirm these initial findings. The role phage cocktails might play in circumventing phage resistance in the presence of antibiotics warrants further study. Furthermore, the genetic analysis of additional *E. faecium* strains, as well as bacteriophages, would provide further insight into the trends we report here.

In summary, we show that in instances of multidrug-resistant *E. faecium*, bacteriophages in combination with DAP and β-lactams are a promising option for eradicating biofilm-mediated infections and preventing the emergence of antibiotic and phage resistance. Additional complementary studies are indicated to examine the addition of multiple phages in a cocktail, as well as further analysis of phage-antibiotic interactions for their potential for in vivo use.

## 4. Materials and Methods

### 4.1. Bacterial Isolates

A panel of five clinical *E. faecium* strains isolated from patients with bacteremia and selected from the Anti-Infective Research Laboratory library, including those with well-characterized *liaFSR* mutations, were evaluated for further experiments.

### 4.2. Antimicrobial Agents and Media

DAP, AMP, and ERT were obtained commercially from Sigma Chemical Company (St. Louis, MO, USA), and CPT analytical powder was obtained from Allergan Pharmaceuticals (Parsippany, NJ, USA). Tryptic soy broth supplemented with 1% glucose (GSTSB) was used for each 24-hour incubation phase immediately prior to the start of experiments. Brain heart infusion (BHI) broth (Difco, Detroit, MI, USA) with 50 mg/L calcium and 12.5 mg/L magnesium was used for susceptibility testing and time-kill analyses (TKAs). To prepare 0.5 and 1.5% BHI agar, respective weight percentages of agar (Oxoid, Lenexa, KS, USA) were added to the broth. For all experiments with DAP, an additional 25 mg/L of calcium was added to the broth due to dependency of DAP on calcium for antimicrobial activity [47].

### 4.3. Bacteriophage Source and Propagation

*E. faecium* bacteriophage 113 (ATCC 19950-B1) and propagating organism *E. faecium* (ATCC 19950) were purchased commercially from ATCC (Manassas, VA, USA). Phage propagation was completed to yield high-titer stocks for use in resistance testing and TKA. An underlay of BHI agar (1.5%) was poured into square petri plates. Next, a 5 mL overlay of 0.5% BHI agar was briefly combined with 20 µL of an overnight host *E. faecium* bacterial culture containing approximately 10^9^ CFU/mL and poured atop the underlay layer. After the overlay was solidified, 500 µL of purified liquid bacteriophage was spread over top and incubated at 37 °C overnight. The overlay agar was scraped into 3 mL of phosphate buffered saline (PBS) + 10 mM magnesium sulfate and centrifuged at 1000 rpm for 25 min at 4 °C. The supernatant was filtered and stored, covered, at 2–8 °C for experimental use [48,49].

### 4.4. Biofilm Quantification Assay

*E. faecium* strains were proven to produce biofilm by biofilm quantification techniques similar to those previously described [43,44]. In brief, *E. faecium* biofilm was formed in a 96-well flat-bottom microtiter tray by incubation overnight at 37 °C in a shaker incubator. The following day, the tray was rinsed using sterile H_2_O and the biofilm was fixed to the tray overnight. Following this, the samples were treated with crystal violet solution (0.2%) for 30 min to allow the dye to penetrate the biofilm. The excess crystal violet was subsequently removed, samples were washed, and the plates were treated with glacial acetic acid (33%) to re-solubilize the biofilm. Biofilm production was read at OD_560_ and samples were analyzed for production compared to wells containing media alone, which was used as a negative control. Biofilm quantification was corrected by subtracting the average OD_560 of_ the media containing only wells that had been treated the same as the wells with biofilm [50]. High, medium, and low categories were determined in relation to the negative control, where high biofilm production was defined as a 3-fold increase from the negative control, medium biofilm production was defined as a 2-fold increase from the negative control, and low biofilm production was defined as a 0.5-fold or lower increase from the negative control.

### 4.5. Phage Sensitivity Assay

Susceptibility of five *E. faecium* strains against Phage 113 was tested using spot testing, where 10-fold serial dilutions of phage were spotted onto 0.5% BHI overlay plates containing overnight culture of the target bacteria. Plates contained 5 mL of overlay agar, which was briefly mixed with 20 microliters of purified phage onto the bacterial lawn. The plates were incubated overnight following drop testing, and the plaque counts were read the following day [42]. Results were qualified as clear, turbid, or no effect of phage (resistant). Susceptible strains were further evaluated via plaque assay to determine efficiency of plating (EOP) as a ratio of plaque forming units (PFU) of the susceptible organism to PFU’s of the reference organism (*E. faecium* strain ATCC 19950) [42]. Phage susceptibility was classified as high, medium, or low based on plaque-forming unit (PFU) counts, where >10^7^ was defined as high, between 10^3^ and 10^7^ was defined as medium, and <10^3^ PFU/mL was defined as low susceptibility. Phage nonsusceptibility was defined as no visual detection of individual phage plaques and/or no bacterial lawn clearance.

### 4.6. Antibiotic Susceptibility Testing

Minimum biofilm inhibitory concentration (MBIC) values were determined in duplicate using the pin-lid method with similar steps to those described previously using the Calgary Biofilm Device (CBD) [45,51,52]. Briefly, a starting inoculum of 1 × 10^6^ CFU/mL in glucose-supplemented TSB (GSTSB) was placed in a 96-well microtiter plate, covered with a p6-peg lid, then incubated for 18–24 h at 37 °C for *E. faecium* biofilm formation on the pegs. Next, the 96-peg lid was removed, rinsed with sterile PBS to remove planktonic bacteria, and transferred to a separate 96-well microtiter plate containing serial dilutions of a single antibiotic using a broth microdilution (BMD) technique, as described by the Clinical and Laboratory Standards Institute (CLSI), except that brain heart infusion (BHI) was used for BMD instead of Mueller–Hinton broth reports and incubated for 24 h at 37 °C [53,54,55]. Following incubation, the peg lid was removed and the MBIC recorded and defined as the column of wells with the lowest antibiotic concentration and without bacterial growth.

### 4.7. Modified Checkerboard for Antibiotic and Bacteriophage Synergy Screening

Antibiotic and bacteriophage synergy in R497 and HOU503 was determined by analyzing the activity of antibiotics and phage 113 in combination compared to their individual activities using a modified checkerboard assay. Initially, a single antibiotic was serially diluted 2-fold through a 96-well round-bottom microtiter tray as was performed for MIC testing, with a starting concentration of 2xMIC for each antibiotic. A second antibiotic was serially diluted 2-fold in the perpendicular direction in a secondary 96-well tray. Antibiotic dilutions in checkerboards containing phage were applied in the same manner as previously described; however, phage dilutions were performed 10-fold rather than 2-fold to achieve a wide range of MOI. Once dilutions were complete, 50 µL from each dilution well of the secondary tray was deposited into the corresponding wells of the original tray. To the original tray, 50 µL each of broth and isolate bacterial stock was added and the plate was incubated at 37 °C for 18–24 h, and then read at OD_600_. In triple-therapy checkerboards assessing synergy of DAP plus AMP in the presence of constant phage, phage 113 was added to each well, excluding controls, at a subinhibitory concentration of 10^−8^ PFU/mL. Synergy, additive activity, and antagonism were defined as a calculated FIC index of ≤0.5, FIC of 1–4, and >4, respectively [37,56].

### 4.8. Time Kill Analyses

TKA were performed in microwell plates to evaluate antibiotic and phage synergy against biofilm-producing organisms, as previously described [57]. In brief, four 3-millimeter polyurethane beads were placed in each well with 2 mL of 1% GSTSB, inoculated with the test organism, and incubated at 37 °C, allowing biofilm formation. Targeted bacterial starting inoculum for both *E. faecium* strains was 6.5–7 log_10_ CFU/mL. After 24 h of incubation, GSTSB was aspirated from each well and replaced with calcium-supplemented BHI broth. Antimicrobials were added at 0.5× the MBIC values (DAP, ERT) or free physiological peak concentration (AMP free peak = 72 µg/mL, CPT free peak = 13.2 µg/mL), whichever was lower, to simulate sub-inhibitory concentrations. Phage dosing was optimized to a multiplicity of infection (MOI) ratio of 1, which represents the ratio of phage to target organism. Beads were removed with sterile forceps at 0 (prior to adding antibiotic and/or phage), 4, 8, and 24 h to create a growth curve. Beads were washed with 1 mL of 0.9% sodium chloride and processed for 6 min alternating in 1-minute intervals each of vortexing and sonication at 20 Hz (Bransonic 12 Branson Ultrasonic Corporation) to disrupt the biofilm structure and recover bacterial cells. Samples were appropriately diluted with 0.9% sodium chloride to eliminate antibiotic carryover [58]. Samples containing phage were then centrifuged and filtered to separate phage and bacteria for counting. The collected bacterial samples were serially diluted appropriately, plated on BHI agar (easySpiral, Interscience for Microbiology, Saint Nom la Breteche, France) with a detection limit of 10^2^ CFU/mL, and incubated for 24 h at 37 °C; bacterial colonies were counted using a laser colony counter (Scan 1200, Interscience for Microbiology, Saint Nom la Breteche, France). Synergy was defined as a  ≥2 log_10_ CFU/mLkill compared to the most effective agent (or double-combination regimen) alone at 24 h. Bactericidal activity was defined as a  ≥3 log_10_ CFU/mL reduction from baseline.

Combinations that resulted in ≥1 log_10_ bacterial growth in comparison to the least active single agent were considered antagonistic. Single drug/phage exposures in biofilm TKA included DAP, AMP, CPT, ERT, and phage 113. Additionally, combination evaluations were performed with DAP plus each of the non-DAP antimicrobials. Statistical analysis was carried out using SPSS version 21.0 (IBM Corp., Armonk, NY, USA) software. One-way analysis of variance (ANOVA) with Tukey’s multiple comparison test was used to compare changes in CFU/mL between regimens, where significance was considered at *p* < 0.05.

### 4.9. Resistance Testing

Each 24 h TKA liquid sample was tested for the emergence of antibiotic and phage resistance, as previously described [22,42,48,59]. The modified BIM (bacteriophage insensitive mutants) test was used to determine the frequency of spontaneous resistance (FOR) [48]. First, BHI agar underlay was poured into 90-millimeter round plates. Following this, 10 µL of each sample was mixed with 100 µL high-titer phage (at ≥10^9^ PFU/mL) and incubated for 10 min at 37 °C. After incubation, this was briefly mixed with 3 mL of 0.5% BHI agar overlay and poured onto the prepared underlay plates. Plates were then incubated for 24 h at 37 °C and bacterial colonies were counted. After counting, plates were left out at room temperature overnight and counted again at 48 h. FOR was calculated as the number of colonies on each plate divided by the number of bacteria in the replicate for both 24- and 48-hour time points. After 48 hours, a single colony was picked from each plate if available, and sub-cultured in BHI liquid culture for 6–7 h at 37 °C [48].

The double-drop method was used after completion of the culture to verify phage sensitivity [42,48]. Using standard agar plates, 10 µL of high titer phage (10^9^ PFU/mL) was dropped onto 5 µL of sub-cultured bacterial samples. Plates were incubated for 24 h at 37 °C and spots were compared to the negative control (made using PBS instead of high-titer phage). Comparatively, samples were labeled as R (resistant, no visible difference from negative control spot), turbid (plaques formed with EOP of 0.001–0.01; T), or S (clear plaques with EOP of 0.1–1; C) [48].

### 4.10. Phage Quantification

Bacteriophages were quantified using a modified small-drop agar overlay method [42]. A 20 µL sample of reference organism cultured for 16–18 h overnight was briefly suspended with 5 mL of 0.5% BHI agar and poured over a 1.5% BHI agar underlay. This was left to set for 10 min. Following this, 5 µL of 10× serial dilutions of filtered phage sample was spotted over top of the bacterial lawn and incubated overnight at 37 °C. Plaques were counted the following day after 16–18 h of incubation.

## Figures and Tables

**Table 1:** High, medium, and low phage susceptibility were defined as phage counts of >10^7^, between 10^3^ and 10^7^, and <10^3^ PFU/mL, respectively.

Organism	Bacteriophage Susceptibility (PFU/mL Compared to Host) ^a^	Biofilm Quantification (OD Compared to Control) ^b^
R497	High	Medium
HOU503	Medium	High
S447 (55)	Low	None
SF12047 (56)	Low	None
12311 (56)	Low	Low

^a^ Modified small-drop agar method results. ^b^ Crystal violet microtiter plate assay results. PFU, plaque forming units; OD, optical density.

**Table 2:** Phage resistance check for bacteriophage and antibiotics at the end of 24 h exposure.

	Bacteriophage Resistance ^a^	
Regimen	R497	HOU503
Phage	R	R
Phage-DAP	S	R
Phage-AMP	S	R
Phage-CPT	S	R
Phage-ERT	R	R
Phage-DAP-AMP	S	S
Phage-DAP-CPT	S	S
Phage-DAP-ERT	S	R

^a^ R, resistant; S, sensitive; DAP, daptomycin; AMP, ampicillin; CPT, ceftaroline; ERT, ertapenem.

## Data Availability

Not applicable.
